# Cyclin-Dependent Kinase 4 and 6 Inhibitors in Cell Cycle Dysregulation for Breast Cancer Treatment

**DOI:** 10.3390/molecules26154462

**Published:** 2021-07-24

**Authors:** Ni Made Pitri Susanti, Daryono Hadi Tjahjono

**Affiliations:** 1 School of Pharmacy, Bandung Institute of Technology, Jalan Ganesha 10, Bandung 40132, Indonesia; dekpitsusanti@unud.ac.id; 2 Study Program of Pharmacy, Faculty of Mathematics and Natural Sciences, Universitas Udaya, Jalan Bukit Jimbaran, Badung 80361, Indonesia

**Keywords:** cell cycle, CDK, cancer, CDK inhibitors

## Abstract

In cell development, the cell cycle is crucial, and the cycle progression’s main controllers are endogenous CDK inhibitors, cyclin-dependent kinases (CDKs), and cyclins. In response to the mitogenic signal, cyclin D is produced and retinoblastoma protein (Rb) is phosphorylated due to activated CDK4/CDK6. This causes various proteins required in the cell cycle progression to be generated. In addition, complexes of CDK1-cyclin A/B, CDK2-cyclin E/A, and CDK4/CDK6-cyclin D are required in each phase of this progression. Cell cycle dysregulation has the ability to lead to cancer. Based on its role in the cell cycle, CDK has become a natural target of anticancer therapy. Therefore, understanding the CDK structures and the complex formed with the drug, helps to foster the development of CDK inhibitors. This development starts from non-selective CDK inhibitors to selective CDK4/CDK6 inhibitors, and these have been applied in clinical cancer treatment. However, these inhibitors currently require further development for various hematologic malignancies and solid tumors, based on the results demonstrated. In drug development, the main strategy is primarily to prevent and asphyxiate drug resistance, thus a determination of specific biomarkers is required to increase the therapy’s effectiveness as well as patient selection suitability in order to avoid therapy failure. This review is expected to serve as a reference for early and advanced-stage researchers in designing new molecules or repurposing existing molecules as CDK4/CDK6 inhibitors to treat breast cancer.

## 1. Introduction

Cell proliferation is a fundamental biological activity in regeneration, development, and homeostasis. In the cell proliferation process, the cell cycle is strictly regulated by certain mechanisms. Cell division only occurs at the required place and time during development, as well as throughout an individual’s life, and the cell content, including each chromosome, needs to be precisely replicated [1]. Furthermore, the combination of intrinsic factors, including the protein synthesis rate, and extrinsic factors, including mitogenic signals, determines the feasibility of a cell entering the cell cycle through a restriction point. The checkpoints are mechanisms monitoring the accuracy, integrity, and order of all steps in the cell cycle, including appropriate cell size growth, chromosome integrity and replication, as well as accurate mitotic segregation [2,3].

Meanwhile, CDKs (cyclin-dependent kinases) are the cell cycle’s main activators through phosphorylation of the key substrates enhancing mitosis progression and DNA synthesis. CDKs are tightly regulated at both the synthesis and proteolysis levels and are activated by cyclin binding. The binding of small inhibitor proteins, CDK inhibitors (CKIs), negatively regulates CDK activity. Therefore, cooperation between cyclin, CDKs, and CKIs is essential for proper cell cycle progression [3,4].

The cell cycle’s dysregulation is induced by a functional imbalance between oncogenes and tumor suppressor genes promoting uncontrolled cell proliferation and, subsequently, development of cancer [5]. Generally, cancer therapy can be categorized based on its target on one or more hallmark of cancer capabilities, which include sustaining proliferative signaling (EGFR inhibitors), evading growth suppressors (CDK inhibitors), resisting cell death (proapoptotic BH3 mimetics), enabling replicative immortality (telomerase inhibitors), inducing angiogenesis (inhibitors of VEGF signaling), and activating invasion and metastasis (inhibitors of HGF/c-Met) [6].

Endogenous and exogenous factors can uniquely influence each tumorigenic process and modify the biological nature of a given tumor [7,8,9]. These can influence and modify the phenotype of a neoplasm that possesses intrinsically dynamic and interacting components of transformed neoplastic cells and nontransformed cells [10]. Local microenvironments within one individual differ from place to place. Changes in the tissue microenvironment can be regarded as a field of cancer susceptibility [11]. Evidence indicates that diet, nutrition, alcohol, lifestyle, medication, the environment, the microbiome, and other exogenous factors have pathogenic roles and also influence the genome, epigenome, transcriptome, proteome, and metabolome of tumor and nonneoplastic cells, including immune cells [10]. These will result in differences in treatment response between cancer patients.

An increased CDK or cyclin expression, or decreased endogenous CKI levels, have been observed in several cancers [12]. CDKs have become a target for anticancer therapy based on their role in cell proliferation. Meanwhile, in the last decade, CDK4/CDK6 inhibitors have been developed as novel anticancer agents, especially for hormone receptor-positive/human epidermal growth factor receptor-2 negative (HR-positive/HER2-negative) metastatic and advance breast cancer [13].

Palbociclib and ribociclib are two CDK4/CDK6 inhibitors permitted for clinical use in these breast cancer treatments, combined with fulvestrant or letrozole [14,15,16,17]. Furthermore, abemaciclib has been accepted as a single-agent therapy for breast cancer, previously treated by endocrine as well as chemotherapy, and combined with fulvestrant for treatment, after progression on endocrine therapy [18,19]. Meanwhile, the newest CDK4/CDK6 inhibitor, trilaciclib, is being studied in small cell lung cancer (SCLC) and triple-negative breast cancer (TNBC) in a bid to prevent chemotherapy-induced myelosuppression [20,21]. This study therefore discusses the cell cycle’s molecular mechanisms as well as the dysregulation in cancer, CDK structures, and drug complexes, as along with selected CDK inhibitors, including FDA-approved and currently undergoing clinical trial CDK4/CDK6 inhibitors.

## 2. Cell Cycle

The cell cycle comprises G1 (first growth/gap), G2 (second growth/gap), S (synthesis), and M (mitosis) phases. During the synthesis (S) phase, a copy of the cell’s genetic material is generated, while all the cell components are spilt into two identical daughter cells during the M (mitosis) phase. G1 (first growth/gap) and G2 (second growth/gap) are cell preparation phases to enable successful completion of S and M phases, respectively [5,22,23]. Cells are able to leave the cycle and enter G0 (non-dividing, resting state) in the absence of a proper mitogenic signal or the presence of a specific antimitogenic signal, leading to a termination in proliferation. Conversely, cell senescence, an irreversible arrest of the G1 cycle where cells are resistant to growth factor stimulation, occurs [24]. Each phase’s completion is verified in the checkpoints, ensuring accurate segregation and replication of chromosomes into daughter cells and preventing tumorigenesis-triggering genomic instability. Subsequently, cells undergo apoptosis, in cases where cellular damage is detected [3,5,22,23]. In addition, the cell cycle comprises three checkpoints occurring at G1-S (R-point or restriction point), G2-M, and metaphase-to-anaphase transitions [3,25,26].

After mitogenic stimulation, D-type of cyclins is expressed, and this promotes CDK4/CDK6 activation. The CDK4/CDK6–cyclin D complexes mono-phosphorylate Rb, RBL1 (p107), and RBL2 (p130) proteins and partially inactivates Rb. Meanwhile, an E2F inhibitor transcription factor is released, and this promotes cyclin E and cyclin A expression. CDK2-cyclin E complexes in turn fully inactivate Rb through hyperphosphorylation, allowing cells to pass the first checkpoint [5,27,28]. Subsequently, cyclin A activates CDK2 to promote the G2 transition (from S to M phase). Finally, at the interphase’s end, cyclin A activates CDK1 to facilitate the onset of mitosis. In addition, CDK1-cyclin B complexes are formed after degrading cyclin A, responsible for driving the cells through mitosis [5,29].

### 2.1. Cyclin Dependent Kinase (CDK)

**Figure 1:**
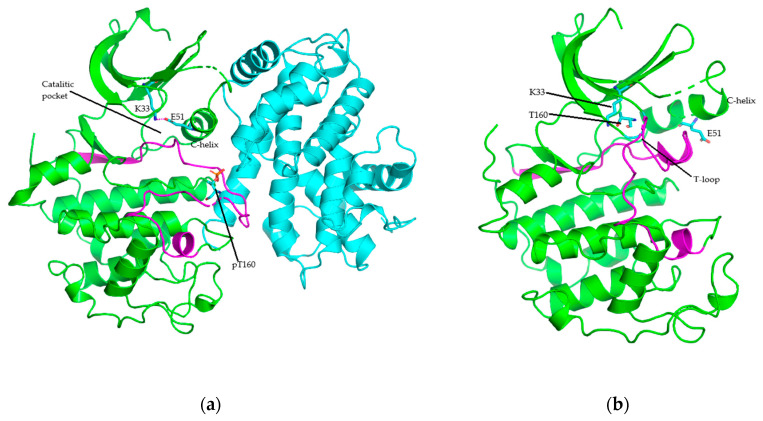
Structure of (**a**) CDK2 (green) bound with cyclin A (cyan) (PDB ID: 4EOQ), and (**b**) monomeric CDK2 (PDB ID: 1HCL). Activation segment in magenta.

In the human cell there are 20 CDKs, and these are threonine/serine protein kinases belonging to the CGMC group based on the kinase domain sequences [30,31]. The first step of CDK activation is the binding with cyclin through hydrophobic interactions between the kinase’s C-helix and a specific helix in the cyclin promoting this segment’s axis rotation. Furthermore, new interactions are generated as a part of the ATP-binding site’s active form. In the next step, cyclin takes the CDK’s C-lobe activation segment out of the catalytic site. Consequently, the conserved threonine residue, T-loop, is accessible for phosphorylation by CDK-activating kinase (CDK7-cyclin H complex) (Figure 1). The CDK heterodimer’s active form is stabilized by this phosphothreonine. Meanwhile, in the monomeric form the T-loop closes the CDK’s catalytic site, preventing enzymatic activity (Figure 1). The C-lobe’s activation segment is also partially disordered [23,32,33,34].

In the CDK4 structure, the CDK–cyclin’s interface differs. Contact between cyclin D and CDK4 only occurs at the N lobe, while contact between cyclin A and CDK2 occurs at the C- and N-lobes. The cyclin binding does not enforce an active CDK4 conformation, and the ATP-binding site remains inaccessible to substrates [35,36]. In addition, phosphorylated CDKN1B protein, a member of CIP/KIPs, allosterically activates the CDK4-cyclin D1 complex and induces structural changes forming the active conformation for catalysis. This protein binding rotates the CDK4 N-lobe relative to the C-lobe, thus releasing the activation segment and allowing substrate binding in the kinase active site. This explains CDKN1B’s necessity in CDK4 phosphorylation by CAK [37,38,39].

### 2.2. Cyclin

Human cyclins comprise 29 proteins with molecular weights between 35 and 90 kDa [23]. Furthermore, cyclin contains five α-helix of 100 amino acid residues called the cyclin box. Group I cyclin (cyclin A, B, D, E, F, G, J, I, and O) contains two cyclin boxes while groups II (cyclin Y group) and III (cyclin C, H, K, L, and T) contain one each [23,24,25,26,27,28,29,30,31,32,33,34,35,36,37,38,39,40]. During the cell cycle, cyclins are expressed in different amounts and degraded by a procedure involving interactions with proteasomes and ubiquitin ligases (E3s). The activation of FOXM1 and E2F transcription factor mediates this expression [5]. In addition, cyclin proteolysis results from the anaphase-fostering complex/cyclosome’s (APC/C) activity during anaphase to the G phase’s end, as well as Skp1-Cul1-F-box (SCF) protein, from the late G1 to the early M-phase [41,42,43,44].

### 2.3. CDK Inhibitors (CKIs)

These include inhibitors of CDK4 and CDK6 (INK4s) as well as the CDK-interacting protein/kinase inhibitory proteins (CIP/KIPs) and are negative regulators of CDK–cyclin complexes. INK4s particularly bind and inhibit CDK6 and CDK4, while CIP/KIPs (CDKN1A, CDKN1B, and CDKN1C) control more of the cell cycle’s aspects through interaction with a wide range of CDK–cyclin complexes [27,45]. Furthermore, CDKN1A is induced through the p53 tumor suppressor protein activity to respond to DNA damage as well as mediate G1 and G2 cell cycle arrests. Meanwhile, CDKN1B accumulates on the cell cycle exit (entering G0 phase) and is rapidly degraded after Ser10 and Thr187 phosphorylation by CDK2 and E3 ubiquitin ligase-mediated degradation as the cell re-enters G1 phase. In addition, CDKN1C, expressed in G0 and G1, inhibits CDK2-cyclin E/A as well as CDK4-cyclin D as a negative cell proliferation regulator. The CIP/KIPs also participate in transcriptional regulation, apoptosis, and cytoskeletal rearrangement [45,46], while the INK4s (CDKN2A, CDKN2B, CDKN2C, and CDKN2D) bind to CDK6 and CDK4, in the G1 phase, thus, preventing cyclin D binding [45,47]. CDKN2A serves as a tumor suppressor agent by delaying the G1 to S-phase transit in cases when the transit is bound to be dangerous to the cell [24]. An enforced INK4 protein expression is also capable of causing G1 arrest by promoting the CIP/KIP proteins’ redistribution and blocking CDK2-cyclin E’s activity [47,48].

## 3. Cell Cycle Dysregulation and Cancer

In several types of cancer, the CDK–cyclin complex’s dysregulation disrupts coordinated cell cycles and promotes uncontrolled proliferation as cancer cells’ characteristic [5,24,49]. Dysregulation occurs due to inappropriate CDK activation involving the cyclin gene’s amplification and protein overexpression, cellular mislocalization or premature cyclin expression, and the CDK–cyclin complex’s activation by preventing the binding of INK4s or CIP/KIPs [5,24,33,50].

### 3.1. Cyclin

Approximately 15–40% of breast, oral, as well as lung carcinomas and melanomas exhibit cyclin D1 amplification [33,50,51]. In addition, increased cyclin D1 expression has been reported in pancreatic, colorectal, head and neck squamous cell, as well as endometrial carcinoma and NSCLC [12,33], while cyclin D1 overexpression or amplification is frequently found in breast cancer [33,50,51]. Meanwhile, cyclin E1 gene amplification has been reported in uterine serous and ovarian carcinomas [52,53]. The level of cyclin E protein expression is associated with increased malignancy in pancreatic, breast, ovarian and colorectal carcinomas, NSCLC, chronic lymphoblastic leukemia, as well as various lymphomas [12,33]. Furthermore, in patients with colorectal adenocarcinoma, cyclin A overexpression is an independent prognostic factor observed in colorectal carcinogenesis and metastasis [33] and is also correlated with less-desirable outcomes in soft tissue sarcomas, endometrial, esophageal, hepatocellular, and thyroid carcinoma cases [12]. In addition, cyclin B mislocalization and/or expression has been reported in primary stages of NSCLC, gastric, colon, thyroid, prostate, esophageal, and breast carcinomas [12,33].

### 3.2. CKIs

Reduced or absent CDKN2A expression has been observed in melanomas, NSCLC, Hodgkin’s lymphomas, retinoblastomas, and osteosarcomas. Deleting the CDKN2B and CDKN2A genes results in decreased expression of these proteins in acute lymphoblastic leukemia, and this is correlated with lower patient survival rate [12,26,54]. In addition, CDKN2A loss in 50–60% metastatic tumors increases the risk of melanoma development [55].

Similarly, reduced or absent CDKN1C expression has been observed in several carcinomas (colorectal, pancreatic, hepatocellular, bladder, and ovarian) and in both childhood as well as adult acute lymphoblastic leukemias [12,26]. Abnormality in CDKN1B expression has also been observed in prostrate, colon, gastric, colorectal, esophageal, as well as breast cancers and gastroenteropancreatic neuroendocrine tumors. These abnormalities are associated with lower survival rates as well as tumor recurrence. Furthermore, diminished survival rate and metastases have been reported in Hodgkin’s lymphomas, gastric, breast, colon, and endometrial carcinomas due to decreased or absent CDKN1A expression [12,26].

### 3.3. CDK

Several lung cancer, diffuse large B-cell melanoma, and lymphoma cases have reported CDK1 overexpression [33,56,57], while CDK2 overexpression has been observed in breast cancer, laryngeal squamous cell cancer, colorectal carcinomas, and melanomas [12,33]. Hyperactivation of CDK2 is correlated with cyclins A and E overexpression and/or amplification in human cancers, particularly lung, thyroid, ovarian, endometrial, and breast carcinoma, osteosarcoma, and melanoma [33]. Furthermore, CDK4 overexpression has been observed in uterine cervical and colorectal carcinomas, melanomas, as well as NSCLC, while CDK4 gene amplification has occurred in glioblastomas, uterine cervix cancer, osteosarcomas, rhabdomyosarcomas, and liposarcomas [12,33]. Overexpression and amplification of this gene has also been reported in sporadic breast carcinomas and melanomas [33,58,59]. Meanwhile, CDK6 overexpression and gene amplification have been reported in leukemias, lymphomas, and gliomas [12]. In several leukemias, translocation of overexpressed CDK6 has the capacity to enhance the interaction between Rb tumor suppressor and p53 pathways in medulloblastoma pathomechanisms [60]. Therefore, high cyclin D expression, low CDKN2A expression, and CDK4 overexpression suggest CDK4 is a potential target for NSCLC, lung, esophageal, colorectal, kidney, pancreatic, liver, breast, prostate, and cervical carcinomas, melanoma, liposarcoma, myeloma, medulloblastoma, mantle cell, and acute lymphoblastic lymphoma treatments [61].

## 4. Structure of Cyclin-Dependent Kinase

**Figure 2:**
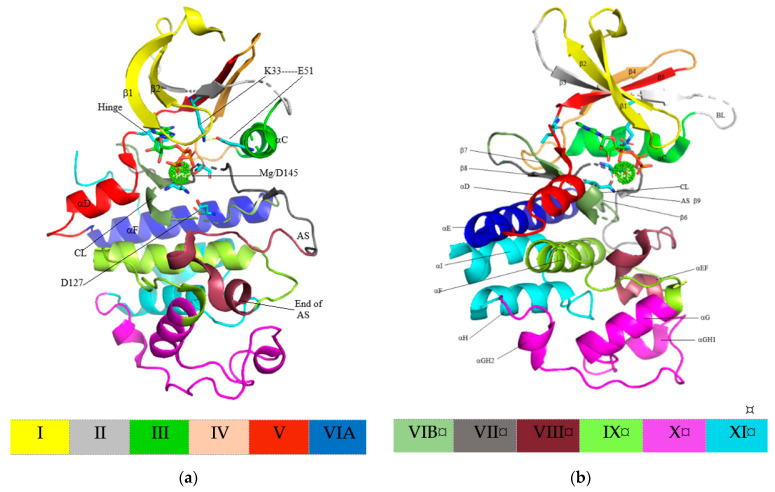
(**a**) Structure of the active CDK2 and (**b**) secondary structure of CDK2 (PDB ID 4EOQ). ATPs are shown in stick format, while Mg^2+^ ions are illustrated as spherical dots. AS: activation segment; BL: back loop; CL: catalytic loop. The twelve domains described by Hanks and Hunter [62] are indicated by the color.

Kinase domains of protein kinase have an important function in protein substrate orientation and binding as well as orientation and binding of ATP phosphate donors forming complexes with divalent cation (Mg^2+^ or Mn^2+^), in addition to γ-phosphate transfer from ATP to the hydroxyl group of the protein substrate’s Ser, Thr, or Tyr residues during catalytic activity [62]. Figure 2 shows twelve kinase domains of the protein kinase primary structure, recognized throughout the superfamily (conserved in over 95% of 370 sequences) and with essential roles in enzyme function. In addition, the kinase domains fold into two-lobed structures [62]. The N-lobe, including subdomains I-IV, comprises five conserved β-strands (β1–β5) primarily involved in nucleotide orientation and anchoring, while the C-lobe, including subdomains VIA-XI, comprises seven conserved α-helixes (αEF and αD–αI) and four short β-strands (β6–β9). These domains are responsible for peptide substrate binding and phosphotransfer initiation. Subdomain V spans two lobes, and a binding between the N- and C-lobes form a deep cleft, known as the catalytic cleft, serving as an ATP-binding pocket [62,63].

CDK catalytic domains comprise 250–300 amino acid residues. Domain I, a gly-rich loop, comprises a GxGxYG signature (^11^GEGTYG^16^ of CDK2, ^13^GVGAYG^18^ of CDK4, and ^20^GEGAYG^25^ of CDK6). This loop occurs between the N-lobe’s most flexible portions, the β1- and β2-strands, accommodating the ATP binding. Domain II in the β3-strand holds a well-preserved AXL sequence (^31^ALK^33^ of CDK2, ^33^ALK^35^ of CDK4, and ^41^ALK^43^ of CDK6), while domain III in the αC-helix comprises a well-preserved glutamate residue. In active protein kinase, this residue generates a salt bridge with the conserved lysine residue (K33-E51 of CDK4, K35-E56 of CDK4, and K43-E61 of CDK6). Domain III in the αC-helix contains the sequence PSTAIRE of CDK2, PISTVRE of CDK4, and PLSTIRE of CDK6, and these interact with the corresponding activating cyclins. Furthermore, conserved HRD sequence in domain VIB is included in the catalytic loop (^125^HRDLKPQN^132^ of CDK2, ^138^HRDLKPEN^145^ of CDK4, and ^143^HRDLKPQN^150^ of CDK6). Meanwhile, the DFG motif in domain VII and conserved APE sequence in domain VIII constitute the activation segment’s beginning and end (^145^DFG^147^-^170^APE^171^ of CDK2, ^158^DFG^160^-^182^APE^184^ of CDK4, and ^163^DFG^165^-^187^APE^189^ of CDK6). This activation segment includes a phosphorylation-sensitive residue called the T-loop (T160 of CDK2, T172 of CDK4, and 177 of CDK6). Meanwhile, the remaining domains form the αF, αG, αH, and αI helixes [12].

**Figure 3:**
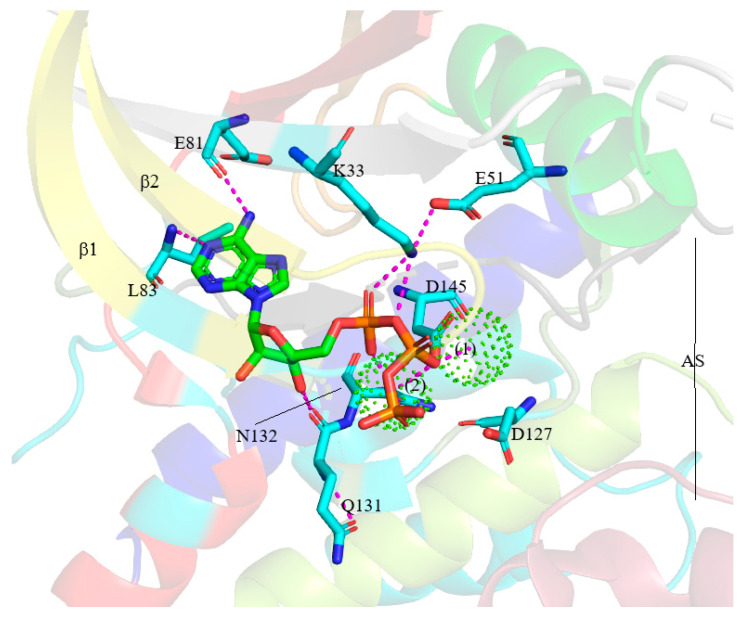
ATP-binding pocket of pCDK2 (PDB ID 1QMZ and 4EOQ). Mg^2+^ ions are shown as spherical dots. AS: activation segment.

The K/E/D/D (Lys/Glu/Asp/Asp) signature motif has the most significant structural and catalytic role of almost all active protein kinases [64]. Furthermore, the lysin (K) and glutamic acid (E) residues are in the N-lobe, while the two aspartic acid (D) residues are in the C-lobe. Figure 1 and Figure 3 show the α- and β-phosphates are held in position by the K33 (the K of K/E/D/D) in the CDK2’s β3-strand. These reactions are stabilized by the salt bridge formed between K33’s ε-amino group and E51’s carboxylate group (the E of K/E/D/D) in the PSTAIRE or αC-helix. This bridge is required for active protein kinase formation and corresponds to the “αC_in_” conformations, in terms of structure [6,58]. However, K33 and E51 failed to form salt bridge in the inactive CDK2, and this corresponds to the “αC_out_” conformation, in terms of structure (Figure 1) [26,64].

A protein/peptide substrate’s binding to the C-lobes of CDK2 involves two Mg^2+^ ions [65]. The D145 residue (the second D of K/E/D/D and the D of DFG) bind with the first ion, coordinating with the ATP β- and γ-phosphates, while the catalytic loop’s N132 residue binds the second ion, coordinating with the ATP α- and γ-phosphates (Figure 3). Subsequently, three hydrophobic contacts are formed between the ATP’s adenine base and A31 of β3 in the catalytic spine, L83 of hinge, as well as L134, after CDK2’s catalytic loop residues. The interaction between most inhibitor molecules and the protein kinase occurs in the ATP-binding pocket, and this is similar to the residues [12]. Furthermore, the N-lobe mobile activation segment’s first residue comprises a DFG (Asp/Phe/Gly) motif. In active protein kinases, the aspartate (D) side chain extends in the ATP-binding pocket’s direction, coordinates the first Mg^2+^ ion, and forms the DFG-D_in_ conformation. Meanwhile, in the inactive counterparts, this side chain extends away from the ATP-binding pocket and forms the DFG-D_out_ conformation [26].

## 5. Protein Kinase–Drug Complex

### 5.1. Binding Pocket within the Catalytic Cleft

ATP binds to the catalytic cleft between the protein kinase’s N- and C-lobes, and this cleft is divided into three regions. The front pocket (FP) comprises a gly-rich loop, hinge residues, an ATP-binding pocket, the extension connecting the C-lobe’s αD-helix to the hinge residues, as well as amino acid residues in the catalytic loop. Front pocket I (FP-I) is found in the solvent-exposed extension connecting the DFG motif and αD-helix to the hinge residues, while front pocket II (FP-II) is found between the gly-rich loop at the C-lobe’s cleft and the β3-strand. Meanwhile, the gate area comprises the activation segment’s proximal portion, including the β3-strand and DFG motif. The back cleft extends to the αC-helix and αE-helix within the C-lobe, portions of the N-lobe’s β4- and β5-strands, as well as the αC-β4 back loop, and forms the back pocket (BP) or hydrophobic pocket II (HPII) together with the gate area [66,67].

The back pocket I (BP-I) occurs between the N-lobe’s DFG-motif, the AXK signature’s conserved β3-K, the C-lobe’s αC-helix, as well as the gate area between the β3- and β4-strands and comprises two subpockets. These are BP-I-A and BP-I-B, located at the gate area’s top and center, respectively. The subpockets both occur in DFG-D_in_ as well as DFG-D_out_ conformations [67].

BP-II-in and BP-II-A-in are found within the DFG-D_in_ conformation’s back cleft, while the BP-II-out region resulted from major changes to BP-II-in and BP-II-A-in, where the DFG-F is present in the DFG-D_in_ conformation. Meanwhile, the BP-II-B region is enclosed by the adjacent αC-helix and β4-strand and occurs in both DFG-D_in_ and DFG-D_out_ conformations. BP-III is found between the conserved catalytic loop HRD-H, αC-helix, activation segment DFG-D_out_, β6- strand, as well as the N-lobe’s αC-β4 back loop, along with the C-lobe’s αE-helix, and is only observed in the DFG-D_out_ conformation. The BP-IV and BP-V pockets are partially solvent-exposed and are found between the C-lobe’s αC-helix, the N-lobe’s DFG-D_out_ motif, the activation segment, β6-strand, and catalytic loop [66].

### 5.2. Classification of Protein Kinase Inhibitors

Protein kinase inhibitors are classified into three types. Type I inhibitors bind to the active kinase’s ATP-binding pocket, while type II bind to the protein kinase’s inactive form. Meanwhile, type III, the allosteric inhibitors, are further divided into type III and IV [68,69]. Type III inhibitors bind in the cleft between the N- and C-lobes, next to the ATP-binding pocket, while type IV inhibitors bind outside the cleft [69]. The other inhibitors type, type I½, bind to the hinge residues, ATP binding pocket, and hydrophobic pocket II in the activation segment’s DFG-D_in_ confirmation, but with αC_out_ conformation [70]. All protein kinases have the capacity to form the DFG-D_out_ conformation, however, not all are capable of forming the αC_out_ conformation [71]. Ribociclib, abemaciclib and palbociclib are αC_out_ CDK4/CDK6 inhibitors approved by the FDA [26]. In addition, type IV inhibitors bind covalently with the target enzyme, while type V are bivalent inhibitors binding to the peptide substrate site and ATP-binding pocket [64,72,73]. The type I½ and type II inhibitors are further divided into A and B subtypes, with only subtype A extending into the back cleft [72].

## 6. Breast Cancer and Treatment

The most frequently diagnosed and most prominent cause of cancer death is breast cancer (BC), followed by lung and colorectal cancer (for mortality), and vice versa (for incidence) in women. The number of new breast cancer cases and deaths in women worldwide is estimated to be 2.08 million and 627,000, respectively [74].

HER2 and hormone receptor (progesterone receptor, PR, and estrogen receptor, ER) are important predictive factors and prognostic markers for anti-HER2-targeted and hormonal therapy. The PR and ER are indicators of responsiveness to hormonal therapy and are expressed in approximately 75% of all breast cancer cases [75,76]. Generally, ER-positive cancers are also PR-positive, with a small number showing single hormone receptor positivity, having higher aggressiveness, and less responsiveness to hormonal therapy [77,78]. HER2 and related genes’ overexpression is observed in about 15% of breast cancers and are associated with poor prognosis, aggressive clinical course, as well as predictive response to anti-HER2 targeted therapy [79]. Meanwhile, the other 10% to 15% of breast cancer cases are TNBC. This type of breast cancer is high degree with poor prognosis and is not efficiently treated by present targeted therapies [80]. Targeted therapy increases life expectancy in breast cancer patients. However, related preclinical studies have shown CDK4/CDK6–cyclin D complex hyperactivity leads to uncontrolled cell proliferation, making the complex’s pharmacological inhibition an interesting therapeutic strategy [81,82].

CDK4/CDK6 inhibitors are most rationally used in ER-positive breast cancer. This type of cancer always retains Rb function, indicating the inhibitor’s major pathway of action remains intact [83]. In addition, CCND1 (encoding cyclin D1), often expressed at high levels in ER-positive breast cancers, is one of the ER’s direct targets. A combination of CDK4/CDK6 inhibitors and standard anti-estrogen therapies showed synergic effect preclinically, and this valuable clinical approach has been confirmed by large randomized clinical trials [14,15,79,80,81,82,83,84,85]. Ribociclib, abemaciclib, and palbociclib are FDA-approved CDK4/CDK6 inhibitors for HR-positive/HER2-negative breast cancer treatment. The combination with endocrine therapy led to improved progression-free survival rates, in patients with this type of breast cancer [14,15,18,19,84,85].

## 7. CDK Inhibitors

These were first developed as first-generation inhibitors called pan inhibitors, and these were relatively non-selective with toxic side effects, suspected to be caused by off-target interactions. Due to these limitations, second-generation inhibitors with greater efficacy, narrower selectivity, and milder side effects were developed [33,61]. A structure elucidation of ATP competitive CDK inhibitors bound to respective targets provided an important hint of the specificity and selectivity profile, as well as the action mechanism [33].

### 7.1. Pan CDK Inhibitors

#### 7.1.1. Flavopiridol/Alvocidib

**Figure 4:**
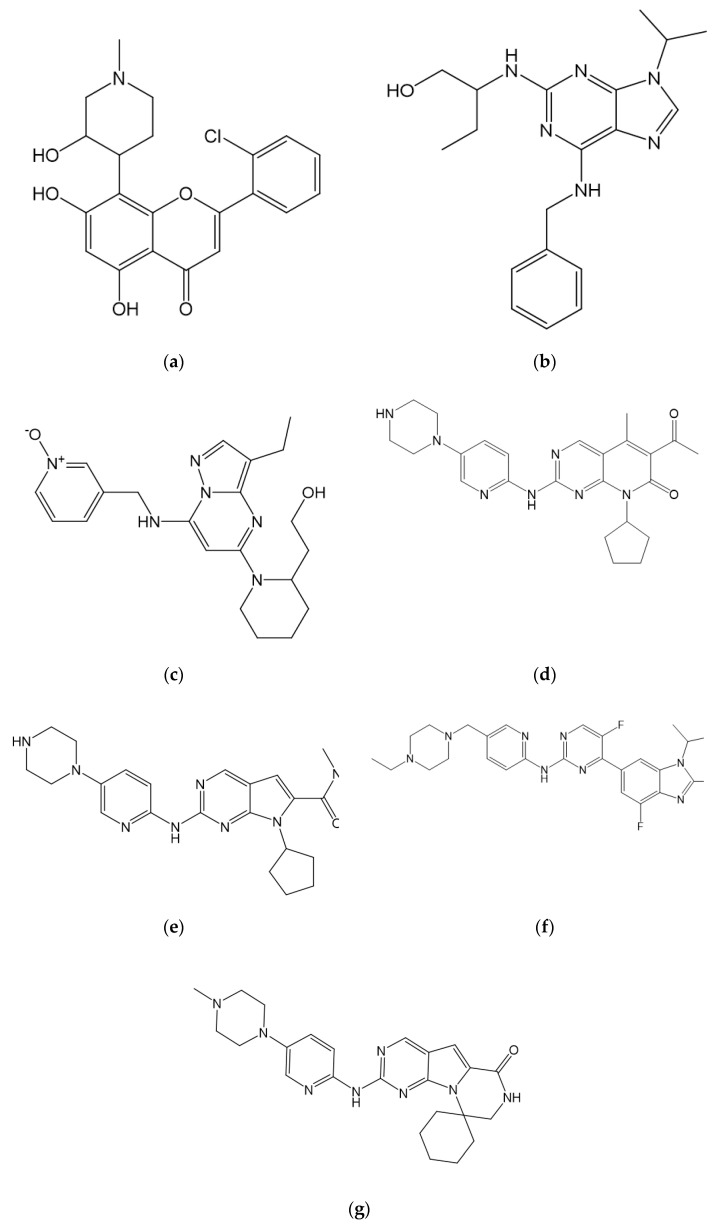
Chemical structure of CDK inhibitors: (**a**) Flavopiridol/Alvocidib, (**b**) seliciclib/roscovitine, (**c**) dinaciclib, (**d**) palbociclib, (**e**) ribociclib, (**f**) abemaciclib, and (**g**) trilaciclib.

Flavopiridol/Alvocidib (2-(2-chlorophenyl)-5,7-dihydroxy-8-[(3S,4R)-3-hydroxy-1-methylpiperidin-4-yl]chromen-4-one) is a piperidine–chromenoce derivative and a semi-synthetic flavonoid (Figure 4) shown to inhibit a broad spectrum of CDKs. Flavopiridol/Alvocidib’s inhibition on cell cycle CDKs (CDK1, CDK2, CDK4, and CDK6) prompts G1 and G2 cell cycle arrest, while inhibition on non-cell cycle CDKs (CDK7 and CDK9) leads to cytotoxic responses due to transcription suppression [61]. Furthermore, Flavopiridol showed significant in vitro activity; however, less activity was observed in vivo [86]. Phase II studies of several solid tumors showed low clinical activity levels, and no phase III studies were conducted. Consequently, the flavonoid’s development was terminated in 2012 [61].

**Figure 5:**
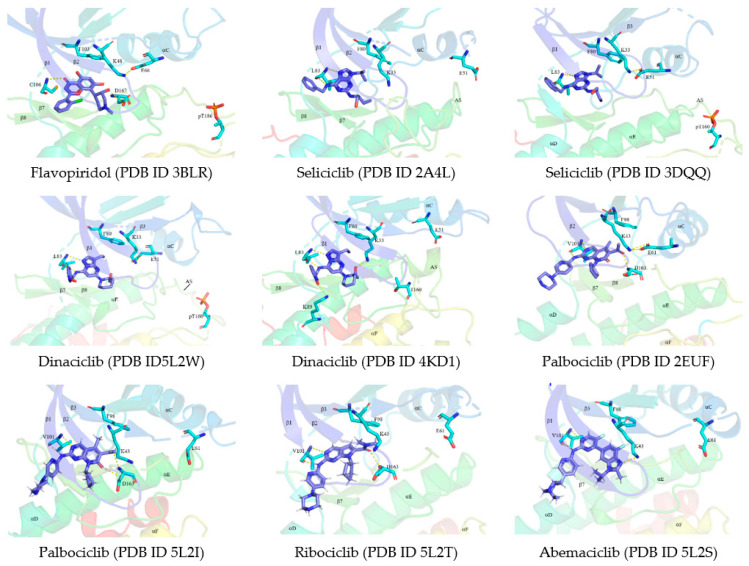
Drug–enzyme complexes and corresponding PDB ID. The drug’s carbon atoms and enzymes are depicted by blue and cyan, respectively, while polar bonds are represented by dashed lines.

Meanwhile, the CDK9–Flavopiridol complex’s X-ray crystal structure (PDB ID 3BLR) shows Flavopiridol located in the cleft between the N- and C-lobes within the ATP-binding pocket beneath the gly-rich loop occurring between the CDK9’s N-lobe’s β1- and β2-strands. Hydrogen bonds are formed between 3-hydroxyl group of the piperidine ring and D167 of DFG and between the chromenone’s C4 carbonyl group and hinge residue’s C106’s N–H group (Figure 5A). Several hydrophobic interactions were also observed on various CDK9 sides, including contact with the β1-strand (I25), gly-rich loop (F30), catalytic spine (V33 and A46), AXK signature (K48), αC-β4 loop (V79), the gatekeeper (F103), hinge residue (F105), catalytic loop (A153 and N154), β7-strand (L156), and the xDFG activation segment (A166 and D167) [26]. Flavopiridol binds to CDK9’s active conformation (αC_in_, DFG-D_in_, and open activation segments) and is grouped as a type I inhibitor [72].

#### 7.1.2. Seliciclib/Roscovitine

Seliciclib/roscovitine ((2*R*)-2-[[6-(benzylamino)-9-propan-2-ylpurin-2-yl]amino]butan-1-ol), a tri-substituted purine, was among the first CDK inhibitors to be evaluated clinically (Figure 4) [61]. According to preliminary data, seliciclib was a relatively specific inhibitor of CDK1, CDK2, and CDK5. However, subsequent data suggests CDK7 and CDK9 were also inhibited, leading to transcription inhibition [87,88]. Several phase I and phase II studies conducted on seliciclib in advanced malignancies, advanced NSLC, and solid tumors showed absent activity with the best response of stable disease and no improvement in progression-free survival [61,87,88]. However, the compound is currently undergoing clinical trials for Cushing disease (https://clinicaltrials.gov/ accessed on 31 May 2021).

Seliciclib’s X-ray crystal structure in complex with inactive CDK2 (PDB ID 2A4L) shows that seliciclib located in the ATP-binding pocket (Figure 5), with the activation segment’s closed conformation, bearing an additional activation segment helix. The β3-strand’s K33 and the αC-helix’s E51 do not form a salt bridge. Hydrogen bonds are formed between the purine base’s N7 and N–H group linked to C3 of seliciclib’s purine base with the N–H and the carbonyl group of CDK2’s hinge residue L83, respectively. Meanwhile, hydrophobic interactions observed on various sides of CDK2 include contact with I10, the catalytic spine (V18, A31, and L134), the β3-strand (K33 aliphatic chain), the gatekeeper (F80), hinge residue (F82), catalytic loop (N132), and A144 (the residue prior to the DFG motif) [12].

In CDK2’s active form, seliciclib is also located in the ATP-binding pocket (PDB ID 3DDQ). CDK2’s activation segment is in open conformation bearing phosphorylated residue pT160. The β3-strand’s K33 and the αC-helix’s E51 form a salt bridge (Figure 5). Furthermore, seliciclib forms similar hydrogen bonds and hydrophobic interactions in both CDK2’s inactive and active conformations, except with K33 and A144 absent in the active CDK2 [12]. Seliciclib is located in CDK2’s front pocket, in both inactive and active conformations [67]. This drug is classified as a type I½ inhibitor, while binding the inactive conformation but as a type I inhibitor while binding with the active conformation [72].

#### 7.1.3. Dinaciclib

Dinaciclib (2-[(2*S*)-1-[3-ethyl-7-[(1-oxidopyridin-1-ium-3-yl)methylamino]pyrazolo [1,5-a]pyrimidin-5-yl]piperidin-2-yl]ethanol), a pyrazolopyrimidine derivative, was specifically developed as a CDK1, CDK2, CDK5, and CDK9 inhibitor with lower activity against CDK4, CDK6, and CDK7 (Figure 4). This drug has superior Rb phosphorylation inhibitory activity compared to Flavopiridol [89]. Based on the phase I study, dinaciclib showed promising results in the form of tolerable toxicity and induced disease stability in a range of malignancies [90]. The result of the Phase I/II single-agent activity trial showed clinical activity in chronic lymphocytic leukemia patients as well as partial response in relapsed multiple myeloma patients, while in the recent phase II trial in solid tumors it showed unsatisfactory results on disease progression [91,92]. Currently, dinaciclib is undergoing clinical trials for several leukemias, hematological malignancies, and breast as well as pancreatic cancer treatments (https://clinicaltrials.gov/ accessed on 31 May 2021).

In addition, dinaciclib is located in the active CDK2–cyclin E complex’s ATP-binding pocket (PDB ID 5L2W). Hydrogen bonds are formed between the compound’s amino group as well as pyrazolo nitrogen and the hinge residue L83 of CDK2’s carbonyl and N–H group, respectively (Figure 5). Meanwhile, hydrophobic interactions occur between the drug and the enzyme, including contact with I10, V18, and A31 of the β1-, β-2, and β3-strands, respectively. The other interactions occur with the αC-β4 loop (V64), gatekeeper (F80), E81, F82, L83, H84, and Q85 (the hinge residues), as well as the catalytic loop’s Q131 and N132, the β7-strand’s L134, and the xDFG activation segment (A144 and D145) [26]. Dinaciclib binds to the CDK2–cyclin E complex’s active conformation (αC_in_, DFG-D_in_, and open activation segments) and is therefore grouped as a type I inhibitor [72].

Dinaciclib’s binding to CDK2’s inactive form (PDB ID 4KD1) shows similar hydrogen bonds and hydrophobic contacts as with the active form. However, an additional hydrogen bond is formed between dinaciclib’s 2-hydroxyl group and K33’s ε-amino group, while a salt bridge is formed between the oxide and K80’s ε-amino group (Figure 5). The enzyme is in an inactive conformation (αC_out_), thus, in this interaction dinaciclib is grouped as a type I½B inhibitor [72], binds within the front pocket of both CDK2 active and inactive conformations, and does not stretch the gate area [67].

#### 7.1.4. Other

AT7519, a dichlorobenzoyl-pyrazole derivative, has shown inhibitory activity against CDK1, CDK2, CDK4, CDK6, and CDK9 [61]. This compound has been undergoing clinical trials for mantle cell lymphoma, chronic lymphoblastic leukemia, non-Hodgkin’s lymphoma treatments, and multiple myeloma (https://clinicaltrials.gov/ accessed on 31 May 2021). Meanwhile, BMS-387032 (also known as SNS-032) was initially found to exhibit higher selectivity toward CDK2 compared to CDK1 and CDK4 but is now also known to target CDK7 and CDK9. However, no further development was reported after phase I clinical trials in selected advanced lymphoid malignancies and tumors [93,94]. The developments of AZD5438 as a potent CDK1, CDK2, as well as CDK9 inhibitor, with lesser selectivity against CDK5 and CDK6, and AG-024322, a potent CDK1, CDK2, as well as CDK4 inhibitor, were ceased due to failure to achieve acceptable clinical results [61,95,96].

### 7.2. Selective CDK4/CDK6 Inhibitors

#### 7.2.1. Palbociclib

Palbociclib (6-acetyl-8-cyclopentyl-5-methyl-2-[(5-piperazin-1-ylpyridin-2-yl)amino]pyrido[2,3-d]pyrimidin-7-one) is the first FDA-approved CDK4/CDK6 inhibitor combined with letrozole for HR-positive/HER2-negative MBC treatment and for treatment of disease progression after endocrine therapy combined with fulvestrant [14,15,17]. The recommended palbociclib dose is a 125 mg capsule taken orally once daily with food for 21 consecutive days, followed by 7 days off treatment (https://www.fda.gov/ accessed on 13 June 2021). Palbociclib is a reversible CDK4/CDK6 inhibitor with a 9–15 nM range of enzymatic half-maximal inhibitory concentration (IC_50_) and is ineffective against CDK1, CDK2, as well as CDK5 at IC_50_ exceeding 10 µM [97].

Furthermore, palbociclib’s core structure is a pyrido[2,3-d]pyrimidinone (Figure 4). Therefore, an excellent combination of potency and selectivity toward CDK4/CDK6 is provided by the cyclopentyl group at the core’s N8. The methyl group at the C5 decreased the inhibition potency toward CDK1 and CDK2 but remained effective against CDK4/CDK6 [98]. Also, the acetyl group addition at C6 maintained selectivity toward CDK4/CDK6 while the 2-aminopyridine addition at the core’s C2 increased in vitro selectivity toward CDK4/CDK6 [99].

Palbociclib’s binding to the complex of CDK6–cyclin V (PDB ID 2EUF) forms three hydrogen bonds, with two of these bonds formed between the palbociclib core’s N3 and the 2-amino group attached to the compound’s pyridopyrimidine core with the N–H group and the carbonyl group of CDK6’s V101 residue. Meanwhile, one hydrogen bond is formed between palbociclib’s 6-acetyl carbonyl group and D163’s N–H group (DFG motif of activation segment). The salt bridge is formed between β3-strand’s K34 and αC-helix’s E61 and the enzyme is in the open activation segment conformation (Figure 5) [12,26]. In addition, the C2 piperazinylpyridinylamino group extends into the solvent, while the 6-acetyl and 5-methyl groups occupy the BP-I-A region in the gate area [67,72].

Also, hydrophobic interaction occurred between palbociclib and I19, V27, A41, and L152 of β1-, β2-, β3-, and β7-strands, respectively. The other interactions occurred on various sides of CDK6, including the αC-β4 loop (V77), AXK signature (K43), the gatekeeper (F98), and H100 (the second hinge residue) proximal to the αD-helix (Q103 and D104), the catalytic loop (Q149 and N150), and A162 (the residue before the DFG motif) [26]. Palbociclib binds to the CDK6–cyclin V complex’s active conformation (αC_in_, DFG_in_, and open activation segment) and is grouped as a type I inhibitor [72].

Furthermore, palbociclib is also bound to inactive CDK6 (PDB ID: 5L2I). The hydrogen bonding patterns and most hydrophobic interactions are identical to the active CDK6 counterpart (Figure 5). The compound binds to the αC_out_ conformation within the front pocket and does not reach the gate area [67]. In this interaction, palbociclib is bound to CDK6’s inactive conformation (αC_out_ and DFG-D_in_) and is regarded as a type I½B inhibitor [72]. Currently, palbociclib is being studied for application in early-stage ER-positive/HER2-negative breast cancer treatment, including adjuvant as well as neoadjuvant studies [100], and is also undergoing clinical trials for non-breast cancer diseases (https://clinicaltrials.gov/ accessed on 31 May 2021).

#### 7.2.2. Ribociclib

Ribociclib (7-cyclopentyl-*N*,*N*-dimethyl-2-[(5-piperazin-1-ylpyridin-2-yl)amino]pyrrolo[2,3-d]pyrimidine-6-carboxamide) is also an FDA-approved CDK4/CDK6 inhibitor combined with a fulvestrant or aromatase inhibitor for ER-positive/HER2-negative advanced breast cancer treatment in postmenopausal women [84,101]. The recommended starting dose of ribociclib is 600 mg orally (three 200 mg tablets) taken once daily with or without food for 21 consecutive days, followed by 7 days off treatment (https://www.fda.gov/ accessed on 13 June 2021). Ribociclib is a highly selective and reversible inhibitor with an IC50 of 10 nM and 40 nM for CDK4 and CDK6, respectively. Most recently, the FDA expanded the inhibitor’s approval to include ER-positive/HER2-negative advanced breast cancer treatment in pre/perimenopausal women [102].

Ribociclib’s core structure is a pyrrolo[2,3*d*]pyrimidine (Figure 4), and three hydrogen bonds are formed in the inhibitor’s interaction with inactive CDK6 (PDB ID 5L2T) (Figure 5). The ribociclib core’s N3 and adjacent amino group N–H form the first and second hydrogen bonds with the N–H group and the carbonyl group of CDK6’s V10 residue, respectively. Meanwhile, the third hydrogen bond is formed between the drug’s carbonyl oxygen and D163’s N–H group (DFG motif of activation segment) [26].

Furthermore, ribociclib forms hydrophobic interactions with enzyme residues, including contact with I19, V27, A41, and L152 of β1-, β2-, β3-, and β7-strands, respectively. The other interactions occur on various CDK6 sides, including the AXK signature (K43), the αC-β4 loop (V77), the gatekeeper (F98), and H99 and H100 (first and second hinge residues), proximal to the αD-helix (D104), the αD-helix (T107), the catalytic loop (N150), and A162 (the residue before the DFG motif) [26]. Ribociclib also binds within the front pocket, FP-I, and the gate area. In this interaction, the inhibitor is bound to CDK6’s inactive conformation (DFG-Din and αCout) and is therefore grouped as a type I½B inhibitor [67,72]. Currently, the drug is undergoing clinical trials for other subtype or breast cancer stages, as well as other cancer types, as a single therapy or combined with other treatments (https://clinicaltrials.gov/ accessed on 31 May 2021).

#### 7.2.3. Abemaciclib

Abemaciclib (*N*-[5-[(4-ethylpiperazin-1-yl)methyl]pyridin-2-yl]-5-fluoro-4-(7-fluoro-2-methyl-3-propan-2-ylbenzimidazol-5-yl)pyrimidin-2-amine) is an FDA-approved CDK4/CDK6 inhibitor with a 2 nM and 10 nM least IC_50_ dosage for CDK4 and CDK6, respectively [103]. The drug is able to last longer on the target compared to palbociclib and is able to cross the blood–brain barrier at low doses, therefore, exhibiting an antitumor effect on metastatic lesions influencing the central nervous system [104,105]. Abemaciclib is FDA-approved as a single treatment as well as in combination with fulvestrant or aromatase inhibitors in HR-positive/HER2-negative metastatic or advanced breast cancer treatment with different clinical settings based on results from several clinical trials [18,19,106]. The recommended starting doses are 150 mg twice daily in combination with fulvestrant or 200 mg twice daily as monotherapy (https://www.fda.gov/ accessed on 31 May 2021).

Furthermore, abemaciclib’s core structure is a pyridine–pyrimidine–benzimidazole compound (Figure 4). The CDK6–abemaciclib complex’s X-ray crystal structure (PDB ID 5L2S) shows three hydrogen bonds are formed with two bonds formed between the carbonyl group as well as the N–H group of CDK6’s V101 and the adjacent amino group N–H, as well as N1 of abemaciclib’s pyrimidine fragment, respectively (Figure 5). Meanwhile, a hydrogen bond is formed between K43’s ε-amino group in the β3-strand and the N1 of abemaciclib’s benzimidazole [26].

The hydrophobic interactions between abemaciclib and CDK6 include contacts with I19, V27, A41, and L152 of β1-, β2-, β3-, and β7-stands, respectively, while other contacts occur in various sides of CDK6, including the AXK signature (K43), the αC-β4 loop (V77), the gatekeeper (F98), H99 and H100 (first and second hinge residues), proximal to the αD-helix (D104), the αD-helix (T107), and A162 (the residue before the DFG motif) [26]. Abemaciclib is located within the FP-II and front pocket, does not extend into the gate area, is bound to CDK6’s inactive conformation (αCout and DFG-Din), and is therefore grouped as a type I½B inhibitor [67,72]. Currently, the drug’s efficacy in other breast cancer subtypes and stages, as well as other cancer types, are being explored, by a number of recruiting and ongoing clinical trials (https://clinicaltrials.gov/ accessed on 31 May 2021).

#### 7.2.4. Trilaciclib

Trilaciclib (4-[[5-(4-methylpiperazin-1-yl)pyridin-2-yl]amino]spiro[1,3,5,11-tetrazatricyclo[7.4.0.0^2,7^]trideca-2,4,6,8-tetraene-13,1′-cyclohexane]-10-one), a pyrido[2,3-d]pyrimidinone byproduct (Figure 4), serves as a very potent and selective CDK4/CDK6 inhibitor. This compound is known to preserve hemopoietin stem and progenitor cells (HSPC) as well as enhance antitumor immunity during chemotherapy [107]. In addition, the proliferation of both lymphocyte and HSPC are dependent on CDK4/CDK6 activity, where these substances are captured in the G1 phase on trilaciclib exposure [108,109,110].

In addition, trilaciclib varies from other FDA-endorsed CDK4/CDK6 samples in terms of its short half, dosing schedule, intended use, and administrative route. These confirmed inhibitors are orally and constantly dosed to prevent the proliferation of CDK4/CDK6-dependent tumors, while trilaciclib is dispensed intravenously to target regular lymphocyte and HSPC populations associated with chemotherapy [110]. Moreover, trilaciclib exhibits significant potential in defending the bone marrow from the cytotoxic impacts of chemotherapy while enhancing immune activity in patients with TNBC, as well as possibly improving both antitumor safety and efficacy [107]. Unfortunately, the X-ray crystal structure binding CDK4 or CDK6 has not been reported. Currently, trilaciclib is undergoing a clinical trial in a patient with metastatic TNBC and SCLC (https://clinicaltrials.gov/ accessed on 31 May 2021).

#### 7.2.5. SHR6390

SHR6390, a novel CDK4/CDK6 inhibitor, demonstrates high potency in in vitro antiproliferative action toward the spread of Rb-positive human tumor cells. It also induces cellular senescence and G1 cell cycle capture, but exclusively reduces Ser780-phosphorylated RB protein levels. However, a significant improvement or equivalent tumor efficacy toward a panel of carcinoma xenograft models compared with palbociclib is observed during oral administration. Furthermore, SHR6390 is believed to resist HER2-targeting antibodies and endocrine remedies in HER2-positive and ER-positive breast cancers, respectively. This success is also recorded in drug resistance to tamoxifen or trastuzumab, and combined with endocrine therapy, greatly exhibits synergistic effects toward ER-positive breast cancer [111]. In advanced cases, SHR6390 showed an adequate safety profile and dose-dependent plasma contact with the recommended phase II dose of 150 mg [112]. Therefore, SHR6390 combined with fulvestrant is currently in a phase III trial for HR-positive/HER2-negative advanced breast cancer (https://clinicaltrials.gov/ accessed on 31 May 2021).

### 7.3. Other Novel CDK Inhibitors

Several novel CDK inhibitors were detected in addition to a detailed knowledge of CDK roles in various breast cancer subtypes, including the results of using CDK4/CDK6 inhibitors in HR-positive/HER2-negative metastatic and advanced breast cancers, with the emergence of resistance and the side effects of the present inhibitors [113]. Panduratin A opposes the G0/G1 phase progression by declining CDK4 and cyclin D1 activities in a dose-dependent manner [114]. Moreover, a flavone compound from *Urginea indica* bulbs tends to stimulate apoptosis, G0/G1phase arrest, and also inhibits angiogenesis in breast cancer cells by restricting CDK1 and CDK6 [115]. In addition, galangin promotes apoptosis by downregulating CDK1, CDK2, and CDK4, resulting in cell cycle capture [116]. Piperlongumine extracted from pepper induces G2/M phase apprehension and opposes CDK1 and CDK4/CDK6, responsible for impeding tumorigenesis in ER-positive breast cancer [117]. Meanwhile, icariin from epimedium brevicornum maxim promotes cell cycle arrest of tamoxifen-resistant MCF-7/TAM cell lines by decreasing CDK2 and CDK4 [118]. Furthermore, resveratrol is believed to target miR-122-5 and influence CDK2, CDK4, and CDK6, leading to a cell cycle capture in addition to improving chemotherapy sensitivity [119]. However, a monoterpenoid β-thujaplicin induces G0/G1 phase arrest and regulates cyclin D1, cyclin E, and CDK4, thereby preventing the ER-basal-like MCF10DCIS.com human breast cancer cell proliferation [120].

## 8. CDK4/CDK6 Inhibitor Combinations

### 8.1. Combination with PI3K-mTOR Inhibitors

The crosstalk between the PI3K-mTOR and CDK4/CDK6 paths provides a strong rationale in combining both routes to inhibit tumor growth [21,85,121]. However, the activation of a compensatory PI3K non-canonical CDK2–cyclin D1 pathway, resulting in Rb phosphorylation, possibly influences the resistance to the CDK4/CDK6 inhibitors in ER-positive breast cancer cell lines. The combination of CDK4/CDK6 and PI3K treatment in these lines tends to surpass the resistance to single-agent CDK4/CDK6 by cyclin D1 downregulation [21,122]. Moreover, the combination of endocrine therapy (fulvestrant), PI3K, and CDK4/CDK6 inhibitors was more effective both in vitro and in vivo compared to either blend of two agents [122]. Furthermore, synergistic interaction between these inhibitors has been also observed in TNBC. This synthesis showed sufficient efficacy, in terms of apoptosis and cell cycle capture, compared to the single drug [123,124].

Cancer cell responsiveness to palbociclib is increased by inhibiting the PI3K signaling pathway through the suppression of post-mitotic CDK2 [123]. Consequently, the fusion of fulvestrant, ribociclib, and a PI3K inhibitor has been investigated in ER-positive/HER2-negative breast cancer. In addition, the current clinical trials explore a combination of CDK4/CDK6 inhibitors, endocrine therapy, and mTOR inhibitor in patients with progress on CDK4/CDK6 inhibitors (https://clinicaltrials.gov/ accessed on 31 May 2021).

### 8.2. Combination with Immune Checkpoint Inhibitors

CDK4/CDK6 inhibitors possibly instigate an antitumor immune response by the stimulation of the T lymphocyte activation effector, reduce T cell proliferation, and enhances tumor cell antigen presentation. The improved immune response primarily involves the immune checkpoints pathway [125,126]. Synergistic inhibition of tumors has resulted in a combination of death protein-1 (PD1) blockade and CDK4/CDK6 inhibitors. Moreover, the fusion of immune checkpoint inhibitors (targeting CTLA-4 and PD-1), PI3Kα, and CDK4/CDK6 inhibitors are known to induce complete and durable regressions in established xenograft mouse models of human TNBC [124].

In patients with advanced cancer, immunotherapy serves as the major treatment to improve outcomes. Superior biomarkers are required to predict therapy results. However, in ER-positive/HER2-negative MBC, a phase IB clinical trial of abemaciclib and an anti-PD1 antibody, pembrolizumab, showed a generally acceptable safety profile with a numerically higher transaminase elevation rate compared to individual treatment. In comparison to historical data for abemaciclib monotherapy in a similar patient population, a numerically higher but vaguely varied overall response rate, progression-free survival, and overall survival was observed. However, further clinical trials are required to provide an absolute description of the combination efficacy in advanced breast cancer patients [127,128].

## 9. Conclusions

Cell cycle progression is described as a complex mechanism involving phosphorylation and dephosphorylation catalyzed by protein kinases and other phosphoprotein phosphatases. The development of potential anticancer agents generally focuses on inhibiting the cell cycle due to the occurrence of dysregulation of cell cycle progression in cancer cells as CDKs and cyclins serve as important regulators. Therefore, CDKs have been interesting pharmacological targets in anticancer drug development since the late 1980s. For more than two decades they were required for the invention of the currently approved CDK4/CDK6 inhibitors. These materials remain an interest for further research regarding high potential benefits in various hematological malignancies, solid tumors, and other diseases. In addition, FDA-approved CDK4/CDK6 inhibitors, including ribociclib, abemaciclib, and palbociclib, as well as other CDK antagonists, are presently in clinical trials as single agents or combined with other drugs for various cancer types. A major challenge in cancer therapy is the high resistance to both non-targeted and targeted drugs. Therefore, the design of vital protocols to minimize or overcome resistance appears to be one of the most important issues in the development of new compounds for future cancer treatment.

## Data Availability

Not available.
